# Changes in growth performance, nutrient digestibility, immune blood profiles, fecal microbial and fecal gas emission of growing pigs in response to zinc aspartic acid chelate

**DOI:** 10.5713/ajas.19.0057

**Published:** 2019-07-01

**Authors:** Yang Jiao, Xinran Li, In Ho Kim

**Affiliations:** 1 Department of Animal Resource and Science, Dankook University, Cheonan, Chungnam 31116, Korea; 2 Jiangsu Key Laboratory of Marine Bioresources and Eco-environment, Jiangsu Ocean University, Jiangsu 222005, China; 3 Department of Mathematics and Statistics, Huazhong Agricultural University, Wuhan 430070, China

**Keywords:** Zinc Aspartic Acid (Zn-ASP), Growth Performance and Nutrient Digestibility, Immune Blood Profiles, Fecal Microbes, Growing Pigs

## Abstract

**Objective:**

This study was conducted to investigate the effect of zinc aspartic acid chelate (Zn-ASP) on growth performance, nutrient digestibility, blood profiles, fecal microbial and fecal gas emission in growing pigs.

**Methods:**

A total of 160 crossbred ([Landrace×Yorkshire]×Duroc) growing pigs with an initial body weight (BW) of 25.56±2.22 kg were used in a 6-wk trial. Pigs were randomly allocated into 1 of 4 treatments according to their sex and BW (8 replicates with 2 gilts and 3 barrows per replication pen). Treatments were as follows: i) CON, basal diet, ii) TRT1, CON+0.1% Zn-ASP, iii) TRT2, CON+0.2% Zn-ASP, and iv) TRT3, CON+0.3% Zn-ASP. Pens were assigned in a randomized complete block design to compensate for known position effects in the experimental facility.

**Results:**

In the current study, BW, average daily gain, and gain:feed ratio showed significant improvement as dietary Zn-ASP increased (p<0.05) in growing pigs. Apparent total tract digestibility (ATTD) of dry matter was increased linearly (p<0.05) in pigs fed with Zn-ASP diets. A linear effect (p<0.05) was detected for the Zn concentration in blood with the increasing levels of Zn-ASP supplementation. Lactic acid bacteria and coliform bacteria were affected linearly (p<0.05) in pigs fed with Zn-ASP diets. However, no significant differences were observed in the ATTD of nitrogen, energy and Zn. And dietary Zn-ASP supplementation did not affect fecal ammonia, hydrogen sulfide and total mercaptans emissions in growing pigs.

**Conclusion:**

In conclusion, dietary supplementation with Zn-ASP of diet exerted beneficial effects on the growth performance, nutrient digestibility, blood profiles and fecal microbes in growing pigs.

## INTRODUCTION

Zinc (Zn) is an important trace element involved in forming more than 300 kinds of metalloenzymes that affect biochemical processes of the whole body, the functions of Zn include regulating immune response, improving gastrointestinal digestion, and interacting with the nervous system [1]. The Zn should be supplemented to most diets to meet the pig’s requirements because of the poor availability of Zn in plant feed ingredients (caused by the binding of Zn with phytate). However, there are great concerns regarding heavy mineral concentrations of agricultural fertilization with the overdose of inorganic Zn [2]. The use of organically complexed or chelated minerals in premixes for livestock diets has been suggested based on the hypothesis that such mineral complexes have a higher bioavailability than inorganic salt analogues [3]. This implies that chelated minerals can be utilized at a much lower concentration in the diet than inorganic minerals without a negative impact on production performance [3]. There were some studies indicated that chelated Zn had positive effects on growth performance, nutrient digestibility, intestine function and immune function in swine [2,4–6] and poultry [3,7,8].

The Zn amino acid chelate is a relatively stable Zn structure. Zn ions are embedded in the middle of two amino acids, and then Zn is transported to the blood through the amino acid channel, so that Zn and amino acids are absorbed by the body, and the absorption rate is greatly improved. Aspartic acid (ASP) is an alpha amino acid that is a common precursor for the synthesis of threonine, lysine, methionine and isoleucine. Gutierrez et al [9] found that the amino group at the end of the polyaspartic acid and the carboxylic acid functional groups on the side chain can combine with metal ions to make the metal ions more stable. Meanwhile, Zn has been reported to increase aspartate aminotransferase activity in fish and mammal plasma [10]. We hypothesized that ASP as an amino acid chelate is a good method to supplement Zn, as after Zn is absorbed by body, its carrier ASP is also absorbed by body to supplement amino acids. As we know, few studies were reported on the application of ASP-metal complexes in animal production. The objective of this study was to investigate the effects of Zn-ASP chelate on growth performance, nutrient digestibility, blood profiles, fecal microbial and fecal gas emission of growing pigs.

## MATERIALS AND METHODS

All animal care and handling procedures used in this study were approved by the Animal Care and Use Committee of Dankook University (Cheonan, Choongnam, South Korea) (DKUIACUC; DKU-3-1710).

### Animals, housing, and experimental design

A total of 160 crossbred ([Landrace×Yorkshire]×Duroc) growing pigs with an initial body weight (BW) of 25.56±2.22 kg were used in a 6-wk trial. Treatments were randomly assigned to pens within gender using the random number generator in excel. Pens were assigned in a randomized complete block design to compensate for known position effects in the experimental facility. After randomization of treatments within gender was completed, average initial BW for each treatment was checked to make sure it was equal (8 replicates with 2 gilts and 3 barrows per replication pen). Treatments were as follows: i) CON, basal diet, ii) TRT1, CON+0.1% Zn-ASP, iii) TRT2, CON+0.2% Zn-ASP, and iv) TRT3, CON+0.3% Zn-ASP. The Zn-ASP was obtained from a commercial company (BTN Co., Asansi, Korea). The mass fraction of ASP that used in this experiment was 95%. Zn-ASP chelate containing 35% of Zn, and Zn^2+^ covalently bound to ASP at molar ratio 1:2. The content of Zn in basal diet was 179 mg/kg, and all diets were formulated to meet or exceed National Research Council (2012) [11] nutrient requirements for growing pig (Table 1). Corn was replaced by Zn-ASP on an equal percent basis. Pigs were housed in partially slatted and concrete floor pens with a pen size of 2.8×5.0 m^2^. All pigs were allowed *ad libitum* access to feed and water through a self-feeder and nipple drinker throughout the experimental period. All pigs were housed in an environmentally controlled room. The target room temperature and humidity were 25°C and 60%, respectively.

### Experimental procedures, measurements, and analyses

Pigs were weighed individually, and feed consumption was measured at the end of the experiment to calculate average daily gain (ADG), average daily feed intake (ADFI), and gain to feed ratio (G:F). For calculations, all data were expressed per pen and per day with average of five pigs/pen in the experiment. The total feed intake for calculating the growth performance was estimated by dividing ADG of each pen by the G:F measured in the total pigs in pens.

Chromium oxide was added to the diet as an indigestible index at 0.20% of the diet for 7 d prior to fecal collection at the sixth week for calculation of the apparent total tract digestibility (ATTD) of dry matter (DM), nitrogen (N), and gross energy (GE). On d 42, fecal samples were collected at random from at least two pigs in each pen (one gilt and one barrow; 16 pigs per treatment). All fecal samples were stored immediately at −20°C until analysis. And fecal samples were freeze-dried and finely ground to be able to pass through a 1 mm screen. The DM, N, and energy were conducted in accordance with the methods established by the Association of Official Analytical Chemists (AOAC 2000). Chromium levels were determined via UV absorption spectrophotometry (UV-1201, Shimadzu, Kyoto, Japan) and the ATTD of DM, N, and energy were calculated using indirect methods described by Williams et al [12]. The digestibility was then calculated using the following formula: digestibility (%) = (1−[(Nf×Cd)/(Nd× Cf)])×100, where Nf, nutrient concentration in feces (% DM); Cd, chromium concentration in diet (% DM); Nd, nutrient concentration in diet (% DM); and Cf, chromium concentration in feces (% DM). Samples were wet acid digested using nitric acid and hydrogen peroxide before the determination of mineral Zn concentration by Inductively Coupled Plasma-Optical Emission Spectroscopy using a Perkin Elmer OPTIMA 7300 (Perkin Elmer Inc., Waltham, MA, USA).

Blood samples were acquired from the cervical vein into both tripotassium ethylenediaminetetraacetic acid (K_3_EDTA) vacuum tubes and clot activator vacuum tubes (Becton-Dickinson Vacutainer Systems, Franklin Lakes, NJ, USA) from 2 pigs (1 gilt and 1 barrow) in each pen. The blood samples were then centrifuged at 3,000×g at 4°C for 15 min within 1 h of collection to separate the plasma. Whole blood samples were analyzed to determine the white blood cell (WBC), red blood cell (RBC), and lymphocyte concentrations using an automatic blood analyzer (ADVIA 120; Bayer, Tarrytown, NY, USA). Immunoglobulin G (IgG) concentrations were evaluated using commercially available enzyme-linked immunosorbent assay kits and analyzed by microplate reader (VersaMax, Molecular device, Sunnyvale, CA, USA). The Zn content in the serum samples was estimated by an atomic absorption spectrophotometer (Thermo Scientific M5 AA Spectrometer, Fremont, CA, USA) after being wet digested with nitric acid via microwave.

Fecal microbes, including lactic acid bacteria and coliform bacteria were determined on fresh morning fecal samples via massaging the rectum from 2 pigs (1 gilt and 1 barrow) per pen at the end of the experiment. One gram of the fecal sample from each pen was diluted with 9 mL of 10 g/kg peptone broth (Becton, Dickinson and Co., USA) and homogenized. Then, 10-fold dilutions of fecal samples were performed (ranging from 10^−1^ to 10^−7^) and then cultivated onto MacConkey agar plates (Difco Laboratories, Detroit, MI, USA) for the enumeration of coliform bacteria and lactobacilli medium III agar plates (Medium 638; DSMZ, Braunschweig, Germany) for the enumeration of lactic acid bacteria. The MacConkey agar plates were incubated for 24 hours at 37°C. The lactobacilli medium III agar plates were then incubated for 48 hours at 39°C under anaerobic conditions. The lactic acid bacteria and coliform bacteria colonies were counted immediately after removal from the incubator. Concentration of microflora was finally expressed as log^10^ colony-forming units per gram of excreta.

At the end of the experiment (d 42), fresh fecal samples were collected from each pen, and fresh urine was collected in a bucket via a funnel below the cage from 8:00 am to 12:00 am. Feces and urine samples were mixed well within a pen and then 300 g subsamples of slurry (150 g feces and 150 g of urine were mixed well, 1:1 on wet weight basis) were stored in 2.6-L sealed plastic boxes in duplicates for the analysis of ammonia, hydrogen sulfide, and total mercaptan emissions. Each box had a small hole in the middle of one side wall that was sealed by an adhesive plaster and then samples allowed to ferment for a period of 7 d at 28°C. The concentrations of ammonia, hydrogen sulfide and total mercaptan were determined at the end of the fermentation period. The gas was sampled and analyzed using a gas sampling pump (model GV-100, Gastec Corp., Kanagawa, Japan; Gastec detector tube No. 3La for ammonia; No. 4LK for hydrogen sulfide; No. 70 for total mercaptans). Before measurement, the slurry samples were manually shaken for approximately 30 s to disrupt any crust formation on the surface of the slurry sample and to homogenize them. The adhesive plaster was then punctured, and 100 mL of the headspace air was sampled approximately 2.0 cm above the excreta surface. Two samples from each pen were measured and then the average was calculated.

All feed samples were analyzed for phosphorus and calcium by the procedure of the AOAC (2000). The lysine content was analyzed by Sykam Amino Acid Analyser (Laserchrom HPLC Laboratories Ltd. Inc., Rochester, UK) after acid hydrolysis for 24 h in 6 mol/L HCl (AOAC, 2000). Nitrogen was determined (Kjeltec 2300 Nitrogen Analyzer; Foss Tecator AB, Hoeganaes, Sweden), and crude protein was calculated as N×6.25. The GE was determined using bomb calorimeter (Mode 1241; Parr Instrument Co., Molin, IL, USA).

### Statistical analyses

The statistical analysis was performed by the general linear model procedure of SAS (SAS Inst. Inc., Cary, NC, USA), with the pen as the experimental unit. Pens were assigned in a randomized complete block design to compensate for known position effects in the experimental facility. The model used was Y_ijk_ = μ+t_i_+r_k_+e_ijk_, where Y_ijk_ was an observation on the dependent variable i_j_; μ was the overall population mean; t_i_ was the fixed effect of Zn-ASP addition treatments, r_k_ was the pen as a random effect, and e_ijk_ was the random error associated with the observation i_jk_. Before conducting statistical analysis of the microbial counts, the value was transformed logarithmically. For the analysis of blood metabolites, the individual pig from each pen was used as the experimental unit. Orthogonal comparisons were conducted using polynomial regression to determine linear and quadratic effects of increasing Zn-ASP levels on all measurements. Variability in the data was expressed as the pooled standard error of the means and probability level of p<0.05 was considered significant.

## RESULTS

### Growth performance

Table 2 presents the growth performance observed in current study. The supplementation of Zn-ASP diets led to a significant linear effect in BW, ADG, and G:F (p<0.05). There was no impact of Zn-ASP dietary supplementation on ADFI.

### Nutrient digestibility

Linear effect was observed in the ATTD of DM (p<0.05) digestibility with the increasing levels of Zn-ASP supplementation (Table 3). However, no significant differences were observed in the ATTD of N, energy and Zn.

### Blood profiles

Table 4 depicts the blood profiles of growing pigs with Zn-ASP supplementation. A linear effect was observed in the Zn (p<0.01). However, IgG, WBC, RBC, and lymphocyte levels remained unaffected by Zn-ASP supplementation.

### Fecal microbial and fecal gas emission

There was a linear increase (p<0.05) in fecal lactic acid bacteria counts with increasing levels of dietary Zn-ASP supplementation, and a linear decrease (p<0.05) was detected for lactic coliform bacteria in growing pigs (Table 5). Dietary Zn-ASP supplementation did not affect fecal ammonia, hydrogen sulfide and total mercaptans emissions in growing pigs.

## DISCUSSION

The Zn affects the cell division and differentiation in animals, it also affects the appetite, material digestion and metabolism of pigs, thus affects the growth performance of pigs [13]. The results of this experiment indicated that the supplementation of Zn-ASP diets led to a significant linear effect on growth performance. As far as we know, no similar studies were found regarding biological functions of Zn-ASP in animal production. Previous studies have focused on methionine Zn chelates. These studies indicated that methionine Zn chelate has a positive effect on growth performance, meat quality and intestinal health of pigs [14,15]. The ASP is the common precursor for the synthesis of methionine, while, the effects of Zn with aspartate on pigs are rarely reported. Creech et al [16] founded that pigs fed chelated Zn gained BW more effectively than those of the pigs fed similar concentrations of trace minerals solely from inorganic sulfate forms. Dietary supplementation with ASP have been reported to enhance growth performance in pigs. Li et al [17] reported that dietary aspartate (1.3%) markedly increased ADG but decreased feed:gain ratio (F:G) in the piglets. Meanwhile, ADG in the L-aspartate (1.0%) group was increased and feed conversion ratio in the aspartate group was decreased, as compared with the H_2_O_2_ group in piglets [18]. These findings were inconsistent with other research that indicated aspartate (2.0%) had no improving effects on the growth performance in piglets [19,20]. Li et al [17] reported that dietary supplementation with high dosages of ASP reduced growth performance and diets with low levels of ASP improved growth performance in piglets. In the current study, the results also show that pigs fed with TRT2 diet had more positive results in growth performance than pigs fed with TRT3 diet. Together, we speculated that low doses of ASP may satisfy the nutritional requirements in healthy piglets, while supplementation with excess ASP exhibit a negative effect on growth performance [17]. More studies are needed to explore the inhibition mechanism of high dose ASP on growth performance in pigs.

Cho et al [21] reported that the ATTD of DM, N, and GE was unaffected by ZnO dietary treatments. However, Qian et al [4] reported that the pigs fed dietary chitosan-Zn chelate had higher apparent digestibility of DM than that of the pigs receiving the CON diet. The current results showed that the utilization of DM in the pigs fed the diet supplemented with Zn-ASP source was enhanced. The possible reasons of increased digestibility of DM might be explained as follows: On one hand, ASP is an important source of energy in small intestinal epithelial cells and can be converted into nutrients by dehydroxylation or aminating to be absorbed and utilized by animals. Liu et al [22] showed that aspartate or asparagine supplementation improved intestinal mucosal which may result in improving the immune function of the intestine. Aspartate plays an important role in maintaining the growth performance and intestinal function of the pig [23]. On the other hand, Zn plays an important role in the synthesis of enzymes, which are essential for nutrient digestion. While, in the current study, there was no difference observed in digestibility of Zn. van Riet et al [24] also found that Zn source did not affect fecal Zn excretion in sows. And Revy et al [13] reported that neither the Zn source nor the interaction between Zn source and phytase level significantly influenced the apparent absorption and retention of Zn in weaning pigs. Meanwhile, our result suggests that the improvement of digestibility of DM might be one of the reasons for the increase of growth performance in Zn-ASP treated pigs.

The Zn is known to play a central role in the immune sys tem, and it affects multiple aspects of the immune system, from the barrier of the skin to gene regulation within lymphocytes [25]. However, the relationship between Zn and the immune function is complex. The Zn deficiency affects development of acquired immunity of T lymphocytes, and B lymphocytes by preventing the outgrowth and certain functions, also Zn deficiency can compromise the B lymphocyte development and antibody production, particularly IgG [26]. Serum Zn was considered an indicator to signify Zn level in the pigs, previous studies already indicated that plasma Zn concentration increased linearly with supplemental Zn [27]. Wang et al [28] indicated that 3,000 ppm of ZnO or 150 ppm of encapsulated ZnO supplementation increased serum Zn concentration in weaned piglets. Seal and Heaton [29] reported that addition of ASP to ZnCl_2_, increased the Zn^2+^ uptake which is in line with the result of the current study. However, Ma et al [2] reported that dietary supplementation of ZnSO_4_, chitosan+ZnSO_4_ and Zn chitosan chelate had no effects on the serum IgG level in weaned piglets. In the current study, IgG levels also remained unaffected by Zn-ASP supplementation. In addition, aspartate is conductive to regulating the immune function through the synthesis of arginine and asparagine. In the synthesis of arginine, aspartate helps to maintain an adequate intracellular concentration of arginine in response to immunological challenges [30]. Meanwhile, in this study, the RBC of pigs had a linear trend among the treatment groups. Therefore, we consider that the increase in RBC and Zn content after the Zn-ASP dietary supplementation is associated with positive effects on immune blood profiles in growing pigs.

Intestine gut microbes are actively involved in nutrient metabolism and animal health, the interaction of intestinal microbiota and immune system keeps intestinal homeostasis. Research enunciated that aspartate enhances intestinal integrity and contributes to the modulation of immune responses [31]. Dietary supplementation with ZnO has been reported to reduce the prevalence of post weaning diarrhea, the numbers of bacteria reaching the ileal mesenteric lymph nodes, and mortality attributed to toxigenic *Escherichia coli* (*E. coli*) infection in pigs [32,33]. Cho et al [21] indicated that increased *Lactobacillus* counts were observed on d 14 and d 42 with the dietary supplementation of modified ZnO: 300 ppm modified ZnO (phase 1)/200 ppm modified ZnO (phase 2). In contrast, Hojberg et al [34] reported that dietary doses of 2,500 ppm ZnO-Zn reduced bacterial activity (ATP accumulation) in digesta from the gastrointestinal tracts of newly weaned piglets compared to that in animals receiving 100 ppm ZnO-Zn. The amounts of lactic acid bacteria (MRS counts) and lactobacilli (Rogosa counts) were reduced, whereas coliforms (MacConkey counts) and enterococci (Slanetz counts, red colonies) were more numerous in animals receiving the high ZnO dose. Broom et al [35] also found that high doses of ZnO (3,100 mg ZnO/kg) tended to decrease lactic acid (p<0.1) bacterial translocation to the mesenteric lymph node and had no effect on *E. coli*. The possible reason for the discrepancies among these studies might be the dose and bioavailability of ZnO. In the current study, linear increase and linear decease were detected for lactic acid bacteria and coliform bacteria with the increasing levels of Zn-ASP supplementation indicating the beneficial effects of Zn-ASP on growth performance and DM was probably due to the improved gut health [21]. Yan and Kim [36] also suggested that the improved microbial balance could increase the conversion of feed to body mass, thus increasing the total metabolism of energy and nutrients. Ferket et al [37] suggested that fecal odor and ammonia emission are directly related to nutrient utilization and the intestinal microbial ecosystem. There appears to be little research on the effect of Zn-ASP dietary supplementation on the fecal gas concentration counts of pigs. Zhang et al [38] found the similar results indicating that dietary supplementation with chelate copper and Zn had no effects on fecal noxious gas emission in weaning pigs. From the present experimental results, even the nutrient digestibility and intestinal microbiota were improved by the dietary Zn-ASP supplementation, while there were no positive effects on fecal ammonia, hydrogen sulfide and total mercaptans emissions in pigs. The potential reasons might be the housing environment, nutritional strategies (feeding programs, type and dose of amino acid supplements), etc. [37]. Moreover, several studies suggested that the fecal noxious gas content was related to the digestibility of nitrogen, because the increased digestibility may allow less substrate for microbial fermentation in the large intestine with consequent decrease in the fecal noxious gas content [39], which may support our study where Zn-ASP supplementation increased DM digestibility without any change in N and E digestibility. More studies are needed to explore the effects of Zn-ASP on noxious gas emission in pigs.

## CONCLUSION

In conclusion, dietary supplementation with Zn-ASP of diet exerted beneficial effects on the growth performance, nutrient digestibility, immune blood profiles and fecal microbial in growing pigs.

## Figures and Tables

**Table 1:** Ingredients and chemical composition of experimental diets (as-fed basis)

Items	Diets
Ingredients (%)	
Corn	45.06
Wheat	13.00
Soybean meal	23.00
Rapeseed meal	2.20
Corn-distillers dried grains with solubles	5.00
Calcium hydrogen phosphate	1.06
Limestone	1.00
Salt	0.30
L-lysine SO_4_-51%	0.24
DL-methionine 50%	0.12
L-tryptophan 10%	0.01
L-threonine 98.5%	0.13
Animal fat	5.30
Molasses	3.20
Choline 50%	0.08
Vitamin premix1)	0.15
Mineral premix2)	0.15
Calculated composition (%)	
Crude protein	17.50
Metabolizable energy (MJ/kg)	14.23
Lysine	0.95
Methionine	0.30
Calcium	0.76
Available phosphorus	0.28
Analyzed composition (%)	
Crude protein	17.51
Metabolizable energy (MJ/kg)	14.21
Lysine	0.94
Methionine	0.30
Calcium	0.78
Available phosphorus	0.27

1) Provided per kilogram of diet: 20,000 IU of vitamin A; 4,000 IU of vitamin D_3_; 80 IU of vitamin E; 16 mg of vitamin K_3_; 4 mg of thiamine; 20 mg of riboflavin; 6 mg of pyridoxine; 0.08 mg of vitamin B_12_; 120 mg of niacin; 50 mg of Ca-pantothenate; 2 mg of folic acid; and 0.08 mg of biotin.

2) Provided per kilogram diet: 140 mg of Cu (as copper sulfate); 179 mg of Zn (as zinc oxide); 12.5 mg of Mn (as manganese oxide); 0.5 mg of I (as KI); 0.25 mg of Co (as Co_2_O_3_**·**7H_2_O); and 0.4 mg of Se (as Na_2_SeO_3_**·**5H_2_O).

**Table 2:** Effects of dietary supplementation in Zn-ASP on growth performance in growing pigs

Items	Treatment1)				SEM	p-value	
	
	CON	TRT1	TRT2	TRT3		Linear	Quadratic
Body weight (kg)							
Initial	22.56	22.56	22.56	22.56	0.42	0.177	0.360
Finish	53.62	54.43	55.40	54.76	0.56	0.029	0.096
Overall							
ADG (g)	740	759	782	766	9.57	0.031	0.095
ADFI (g)	1,750	1,766	1,773	1,754	20.50	0.805	0.378
G:F	0.423	0.430	0.441	0.437	0.005	0.027	0.260

Each mean represents 8 replicates (n = 8/treatment).

Zn-ASP, zinc aspartic acid; SEM, standard error of the means; ADG, average daily gain; ADFI, average daily feed intake; G:F, gain to feed ratio.

1) CON, basal diet; TRT1, CON+0.1% Zn-ASP; TRT2, CON+0.2% Zn-ASP; TRT3, CON+0.3% Zn-ASP.

**Table 3:** Effects of dietary supplementation in Zn-ASP on nutrient digestibility in growing pigs

Items (%)	Treatment1)				SEM	p-value	
	
	CON	TRT1	TRT2	TRT3		Linear	Quadratic
DM	74.97	75.87	77.88	76.32	0.60	0.034	0.040
N	74.49	75.05	77.14	75.27	0.95	0.307	0.212
Energy	75.82	76.02	77.58	76.37	0.88	0.421	0.430
Zn	77.46	78.26	79.61	78.80	0.95	0.217	0.529

Each mean represents 8 replicates (n = 8/treatment).

Zn-ASP, zinc aspartic acid; SEM, standard error of the means; DM, dry matter; N, nitrogen; Zn, zinc.

1) CON, basal diet; TRT1, CON+0.1% Zn-ASP; TRT2, CON+0.2% Zn-ASP; TRT3, CON+0.3% Zn-ASP.

**Table 4:** Effects of dietary supplementation in Zn-ASP on immune blood profiles in growing pigs

Items	Treatment1)				SEM	p-value	
	
	CON	TRT1	TRT2	TRT3		Linear	Quadratic
WBC (10^3^/μL)	23.2	22.2	20.3	19.7	2.00	0.192	0.922
RBC (10^6^/μL)	6.4	6.8	7.1	7.0	0.21	0.053	0.213
Lymphocyte (%)	49.6	53.9	54.2	54.7	2.74	0.222	0.504
IgG (mg/dL)	512	520	529	546	16.72	0.170	0.809
Zn (μg/dL)	100	158	177	205	9.84	<0.001	0.160

Each mean represents 8 replicates (n = 8/treatment).

Zn-ASP, zinc aspartic acid; SEM, standard error of the means; WBC, white blood cell; RBC, red blood cell; IgG, immunoglobulin G; Zn, zinc.

1) CON, basal diet; TRT1, CON+0.1% Zn-ASP; TRT2, CON+0.2% Zn-ASP; TRT3, CON+0.3% Zn-ASP.

**Table 5:** Effects of dietary supplementation in Zn-ASP on fecal microbial and fecal gas emission in growing pigs

Items	Treatment1)				SEM	p-value	
	
	CON	TRT1	TRT2	TRT3		Linear	Quadratic
Fecal microbial (log^10^cfu/g)							
Lactic acid bacteria	7.55	7.59	7.72	7.73	0.06	0.021	0.803
Coliform bacteria	6.13	6.07	5.99	5.94	0.07	0.032	0.957
Fecal gas emission (ppm)							
Ammonia	19.0	18.4	18.1	17.6	1.02	0.352	0.953
Total mercaptans	0.6	0.5	0.3	0.4	0.16	0.235	0.486
Hydrogen sulfide	4.9	4.5	4.2	3.9	0.63	0.290	0.969

Each mean represents 8 replicates (n = 8/treatment).

Zn-ASP, zinc aspartic acid; SEM, standard error of the means.

1) CON, basal diet; TRT1, CON+0.1% Zn-ASP; TRT2, CON+0.2% Zn-ASP; TRT3, CON+0.3% Zn-ASP.
